# Digital detection of craving and stress for individuals in recovery from substance use disorder: A qualitative study

**DOI:** 10.1016/j.dadr.2025.100336

**Published:** 2025-04-19

**Authors:** Jazmin Hampton, Reynalde Eugene, Nirzari Kapadia, Emily Caggiano, Amanda Geagea, C. James Watson, Stephanie Carreiro

**Affiliations:** a Division of Medical Toxicology, Department of Emergency Medicine, University of Massachusetts Chan Medical School, 55 Lake Ave N, Worcester, MA 01655, United States; b Division of Medical Toxicology, Department of Emergency Medicine, Maine Medical Center, 22 Bramhall St, Portland, ME 04102, United States

**Keywords:** Substance Use Disorder, Digital Health, Stress, Craving

## Abstract

**Aims:**

This study aims to 1) categorize experiences with stress and craving during substance use disorder (SUD) treatment, 2) explore perceptions of both clients and treatment providers towards a digital detection system for stress and craving during recovery, and 3) identify barriers and facilitators to adopting this technology during SUD treatment.

**Methods:**

This was a qualitative study of people in recovery from SUD (clients) and healthcare providers from outpatient treatment facilities in the northeast United States. Clients were asked to use a digital health tool that detects physiological biomarkers of stress and craving (RAE Health) for 30 days alongside their usual treatment and to engage in a semi-structured interview upon completion. Providers were asked to participate in a one-time focus group.

**Results:**

Thirty-one clients completed a semi-structured interview, and eleven providers participated in two focus groups. Four core themes emerged from the qualitative data: categorization of experiences with stress and craving, perceptions of digital detection systems, perceived barriers and facilitators of the system, and desired features of the system. Overall, client and provider perception were positive, and acceptability of the digital health tool was high.

**Conclusions:**

A digital detection system for stress and craving during SUD recovery was perceived favorably by both clients and providers, with clients citing heightened awareness and providers citing opportunities for personalized care as promising use cases. Future iterations of digital health systems for this population should consider the ideal “dose” of the intervention to maximize benefit.

## Highlights

• Digital biomarkers using wearable sensors can help categorize stress and craving.
• Digital detection systems offer increased awareness and accountability.
• Digital detection systems offer providers an opportunity to personalize treatment.
• The optimal dose of the digital intervention is a critical factor in distinguishing value vs burden.

## 1 Introduction

Approximately 39.5 million people world-wide suffer from SUD (Substance Use Disorder), (SAMHSA, 2023). Even with treatment, relapse rates for those with SUDs are high: data suggest that 50–90 % of those undergoing treatment for SUD return to use. (Chen et al., 2019, Bentzley et al., 2015, Weiss et al., 2011). Additional solutions to augment current treatments are needed to reduce the burden of SUDs and decrease rates of return to use. Mobile health (mHealth) tools facilitate healthcare delivery through mobile devices, including smartphones and wearables (Park, 2016). One systematic review of mHealth for Alcohol Use Disorder (AUD) indicates that mHealth is effective at decreasing alcohol use (Kruse et al., 2022). Another systematic review of digital health interventions found they were associated with reduced substance use and craving (Carreiro et al., 2020b).

One valuable aspect of mHealth is the application of just-in-time-adaptive interventions (JITAI) during times of increasing need. Stress and cravings, two key factors driving return to use, are potential targets for JITAI. Stress is the experience of events deemed by the observer as harmful, challenging, or threatening (Sinha, 2001). Craving is a conscious desire to use a substance of choice (Tiffany and Wray, 2012). Traditionally, assessment of stress and craving in the field has been done via subjective measures, such as self-report. However, recent research has demonstrated that objective digital biomarkers of stress and craving are detectable using physiologic data from wearables (Carreiro et al., 2020a). Although there is growing evidence supporting JITAI for SUD and for digital biomarker-based craving and stress detection (Businelle et al., 2024, Carreiro et al., 2020a, Carreiro et al., 2020b, Perski et al., 2022), there is still limited research on real-world implementation and integration into SUD treatment.

This present study aims to 1) categorize experiences with stress and craving during SUD treatment, 2) explore perceptions of both clients and treatment providers towards a digital detection system for stress and craving during recovery, and 3) identify barriers and facilitators to adopting this technology during SUD treatment.

## 2 Methods

### 2.1 Overall study design

This is a qualitative analysis of semi-structured interviews of individuals in SUD treatment (clients) and treatment center staff (providers). All study-related protocols were approved by the UMass Chan Medical School Institutional Review Board (docket #H00017213), and informed consent was obtained from all participants.

Client interview data were obtained as part of a larger study evaluating a digital tool for stress and craving detection during SUD recovery. Clients were recruited from two private outpatient treatment facilities in the northeast region of the United States specializing in home-based SUD treatment via study flyers and word-of-mouth referrals. Clients were asked to use RAE Health, a digital health tool, for 30 days alongside their usual treatment. Clients completed a semi-structured interview at the end of the 30-day study period. The quantitative results of this study are reported elsewhere (Carreiro et al., 2024).

Providers involved in the clients’ care were recruited for this study via informational staff meeting presentations, and their data were obtained in two focus groups. All providers were familiarized with the RAE Health system through a hands-on demonstration session and/or had prior experience with their clients using the app as part of the parent study. Provider feedback was sought to provide a supplemental perspective on how RAE may contribute to treatment.

### 2.2 RAE (Realize. Analyze. Engage.) Health

The RAE system is a digital health intervention for individuals in recovery for SUD, which consists of a wearable sensor that continuously measures physiology and an interactive, client facing app. Embedded in the app are previously validated machine learning algorithms that use these data to identify biomarkers of stress and craving using physiologic data (i.e. accelerometry, skin conductance, skin temperature, heart rate) (Carreiro et al., 2020a, Carreiro et al., 2021), which then pushes a notification to the client. Upon receipt of a stress notification, clients have the option to input additional contextual data about the event and/or participate in a de-escalation exercise (mindful breathing or journaling). Trend visualizations of stress and craving events over time are available in the app for the client and their provider to review. Clients were encouraged (but not required) to share app data with their providers during routine scheduled treatment visits, which includes visualizations of trends in stress and craving alerts over time and journal entries that coincide with stress/craving events. Clients and providers were also encouraged to use the RAE Health data as a tool to facilitate reflection and identify personal triggers.

### 2.3 Inclusion and exclusion criteria

Clients were included that (1) were ≥ 18 years old, (2) were enrolled in outpatient treatment for SUD, (3) spoke English, (4) had access to an iOS or Android-capable smartphone, and (5) could provide informed consent. Clients were excluded if they: (1) were pregnant, (2) had physical limitations preventing the use of the wearable, or 3) were a prisoner.

Providers were included that (1) were ≥ 18 years old, (2) were employed as a clinician, nurse, case manager, social worker, administrator, or peer recovery professional in an outpatient SUD treatment facility, (3) spoke English, and (4) could provide informed consent. Providers were selected that would engage with the RAE system from a variety of perspectives, including those that would work directly with clients (e.g. clinicians, nurses, recovery coaches) and those that may be involved in the implementation of RAE from a systems perspective (administrators, clinician/peer/nurse supervisors).

### 2.4 Qualitative data collection

Following the 30-day study period, we conducted qualitative semi-structured interviews via phone or video conference (see Supplemental Material for interview guide) with each client to assess their perceptions of stress, craving, and the use of a digital detection system. We examined usability, barriers to initial use, the degree to which interactions were bothersome, and the degree to which continuous physiologic monitoring and/or app interactions affected behavior. We assessed preferences regarding in-app annotation format (e.g. how users added details of stress and craving events), information delivery method, and participant’s interest in using the system for an extended period. Finally, we explored barriers and facilitators of long-term use.

We conducted focus groups with providers to assess their perceptions of and experience with the RAE system (see Supplemental Material for focus group guide). Focus group discussions (conducted via video conference) included clinical utility of gathered data, barriers to use, practice changes (positive or negative) resulting from system use, overall impressions of the system’s contribution to the treatment plan, and additional features that might increase ease of use and adoption.

Semi-structured interviews and focus groups were audio recorded and transcribed verbatim by a HIPAA-compliant professional transcription service.

### 2.5 Data analysis

Applied thematic analysis was used to code and analyze the data. After completing all data collection, the codebook was developed. The initial coding structure was developed using deductive codes from the interview questions. However, after team members familiarized themselves with the data by reading through transcripts and reflection notes, the codebook was also supplemented with inductive codes generated through the review of transcripts. The codebook was developed as a simple table (please see Supplemental Material for codebook). Once the coding scheme was agreed upon, two coders independently coded each transcript on paper. The two coded transcripts were then compared for concordance and completeness. A third team member resolved persistent discrepancies. The finalized codes were uploaded into NVivo qualitative analysis software (Version 12, Lumivero, Denver, CO). Subsequently, emergent themes were identified, explored and summarized.

## 3 Results

### 3.1 Sample characteristics

Sixty clients enrolled in the parent study from December 2019 to May 2021: 31 completed qualitative exit interviews. The remaining 29 clients were either lost to follow-up (N = 28) or declined (N = 1). The final client cohort was 64 % female (mean age 41.5 years, range 23–62). Demographics, including those who did not complete the exit interview, are listed in Table 1. Detailed SUD characteristics of the population who completed the exit interview are listed in Table 2. We conducted two provider focus groups with a total of 11 providers (six in focus group one, and five in focus group two), including case managers (2), peer recovery advisors (6) and administrators (3). Focus group participants were 63 % female (7/11).

**Table 1:** Client demographics (by interview completion status).

	**Did not complete (N = 29)**	**Completed (N = 31)**	**Overall (N = 60)**
**Age**			
Mean (SD)	41.7 (13.3)	41.4 (9.46)	41.5 (11.2)
**Sex**			
Male	14 (48.3 %)	22 (71.0 %)	36 (60.0 %)
Female	15 (51.7 %)	9 (29.0 %)	24 (40.0 %)
**Race**			
Asian	0 (0 %)	1 (3.2 %)	1 (1.7 %)
Black or African American	2 (6.9 %)	0 (0 %)	2 (3.3 %)
White	22 (75.9 %)	29 (93.5 %)	51 (85.0 %)
Other	4 (13.8 %)	1 (3.2 %)	5 (8.3 %)
**Hispanic/Latinx**			
	1 (3.4 %)	2 (6.5 %)	3 (5.0 %)
**Education**			
Some high school (no diploma)	1 (3.4 %)	0 (0 %)	1 (1.7 %)
High school graduate (diploma or equivalent)	6 (20.7 %)	1 (3.2 %)	7 (11.7 %)
Some college credit (no degree)	6 (20.7 %)	6 (19.4 %)	12 (20.0 %)
Associate degree	0 (0 %)	1 (3.2 %)	1 (1.7 %)
4-year college graduate (bachelor's degree)	4 (13.8 %)	7 (22.6 %)	11 (18.3 %)
Graduate or professional degree	0 (0 %)	2 (6.5 %)	2 (3.3 %)
**Phone Type**			
iOS	12 (41.4 %)	24 (77.4 %)	36 (60.0 %)
Android	15 (51.7 %)	5 (16.1 %)	20 (33.3 %)
**Time in treatment**			
0–30 days	3 (10.3 %)	8 (25.8 %)	11 (18.3 %)
1–3 months	7 (24.1 %)	7 (22.6 %)	14 (23.3 %)
3–6 months	3 (10.3 %)	1 (3.2 %)	4 (6.7 %)
6 months−1 year	7 (24.1 %)	1 (3.2 %)	8 (13.3 %)
1–5 years	1 (3.4 %)	4 (12.9 %)	5 (8.3 %)
5 + years	0 (0 %)	3 (9.7 %)	3 (5.0 %)

**Table 2:** Client SUD and treatment characteristics.

**Treatment Substance(s)**		**Type of Treatment(s)**	
	**Overall (N = 31)**⁎		**Overall (N = 31)**⁎
**Alcohol**	25 (80.6 %)	**Outpatient**	25 (80.6 %)
**Opioids**	7 (22.6 %)	**12-step program**	9 (29.0 %)
**Cocaine**	1 (3.2 %)	**MAT/MOUD**	3 (9.7 %)
**Methamphetamine**	1 (3.2 %)	**Sober Living Home**	3 (9.7 %)
**Sedatives**	1 (3.2 %)	**Other**	5 (16.1 %)
**Other stimulants**	1 (3.2 %)		

⁎ Totals are > 100 % as clients could select > 1 substance and/or treatment type

Four core themes emerged from the qualitative data: categorization of experiences with stress and craving, perceptions of a digital detection system for stress and craving, perceived system barriers/facilitators and desired system features.

### 3.2 Theme 1: experiences with stress and craving

Clients described stress and cravings based on timing, context, and impact on recovery. Clients tended to use the terms “stress” and “anxiety” synonymously in discussion. Sources of stress included work or school, scheduling, and relationships with family (specifically parents and young children), friends and partners. Other sources were family members with SUDs, the COVID-19 pandemic, driving, finances, and reintegration into “sober” society. The frequency of notable stress events varied from daily to weekly. Stress decreased over time but never disappeared. Most clients did not explicitly express stress as a major factor impacting their recovery, but many implied that it could lead to return to use. Compared to stress, clients found it harder to define a craving and expressed uncertainty about whether cravings were “real” or “true”. They tended to subdivide cravings into several different categories (e.g. “physical” versus “mental” cravings, “true cravings” versus “coping mechanisms”).

“I believe [craving is] a mental thing too because when I drink…I feel good … you know how you feel like you happy, you wanna do something? It triggers you, like you get like excited to drink….”

– 44-year-old male in recovery from alcohol use disorder

“It was more romanticized than it was a physical craving or a desire of need, if that makes sense…So like I almost feel like that started to become a physical thing, ‘well okay, I'm not gonna be able to relax until I have a drink’ kind of a feeling. I had that in the beginning, which I don't have anymore.”

– 49-year-old male in recovery from alcohol use disorder

Clients reported they had fewer cravings compared to stress events. The most commonly cited triggers included seeing their substance of choice, environmental reminders of their substance of choice, driving, boredom, hunger, and physical withdrawal. The frequency of cravings was variable in the sample, ranging from rare/transient to daily/weekly. Overall, cravings decreased as time from last use increased. Clients also noted that cravings were triggered by old habits associated with substance use (e.g. certain television shows, past experiences/memories, sunny days).

“For cravings, watching television, a commercial or a song that I heard and I, you know, relate to having drinks or being at a party or being at a concert. Those kind of things [triggered me].”

– 47-year-old female in recovery from alcohol use disorder

### 3.3 Theme 2: digital detection system perceptions

Clients generally found the digital detection system to be useful in SUD recovery and were enthusiastic about its potential. Some felt it was beneficial in their recovery, reporting that they felt empowered and worthy of recovery, although others were indifferent. Those who found it beneficial reported that it increased awareness, distracted them from dwelling on stressors/cravings, and created a sense of accountability and empowerment. They reported that real-time data reminded them of their recovery, and it would be helpful if the system could forewarn them of cravings or stress events.

“[the app is] … another tool in my toolbox….and I need all the tools that I can get.”

– 56-year-old male in recovery from alcohol and opioid use disorders

“It was a good experience and if in the future to have something, [to] maybe warn you, or forewarn you when your body sensations are you gonna be triggered or crave.”

– 47-year-old female in recovery from alcohol use disorder

“…part of the recovery process is just awareness in general…and accepting whatever's going on, and then just to have that indicator to try to…deal with some of that stuff. Just the identification of circumstances, things that bothered me that I wouldn't have identified before.”

– 42-year-old male in recovery from opioid use disorder

Clients had varying opinions on when in their course of recovery the system would be of greatest benefit and ultimately agreed that it may differ by individual. Most expect the system to have the greatest benefit early in their recovery but pointed out that it could be an added stressor if used too early in recovery. Clients advocated that its use should follow any detoxification and stabilization period when individuals have established more structure in their lives.

Providers felt strongly about the digital detection system and were eager to implement the system into their practice. Their intended use cases for the system included assessing an individual’s need for additional support, adequacy of the treatment plan, provider fit, and risk level in addition to internal communication and motivation of staff within the treatment program. They envision that this system could help monitor staff’s caseload, response metrics, and provide staff support as needed.

“… [the system could] pick up on the yellow flags before they become red flags. Being able to like check in and say …we’re noticing a lot of cravings, maybe they need a little bit of extra support that day or “let's go to a meeting” …”

*– Focus group provider*

“Anything that you’re doing for your recovery, you could track it cause a lot of that [is] building recovery capital. It’s almost like, from my experience, almost like a snowball like going downhill. Like the more stuff you do, the more stuff you want to do, and you can kind of look back and feel good about it.”

– Focus group provider

### 3.4 Theme 3: barriers and facilitators to adopting RAE Health during treatment for SUD

Facilitators of system use identified by clients included streamlined commercial sensor aesthetics of the sensor and the use of a “buddy system” (e.g. partner, child, clinician, or peer who also participated in the digital detection system). Clients highlighted the use of a small, commercially available smartwatch with a discreet, "normal" appearance as a key benefit, as it did not signal any connection to SUD treatment. They also appreciated that it was easy to use.

Providers noted most clients (and providers) are already using a variety of apps to track recovery metrics, some of which are intended for SUD/mental health and others which are more general and re-purposed for this indication. They noted that intuitive apps that provide a reward are most desirable. They also noted that apps should serve a purpose for clients (e.g. fitness tracking, goal tracking) beyond collecting data for their provider’s benefit.

“You should just be able to open it and know how to use it right away…. with minimal training, maximum satisfaction, and easy to use.”

– Focus group provider

“[The app should] actually give them something and not to just ask them to do stuff for us.”

– Focus group provider

Barriers were divided into two main categories: craving/stress management barriers and app/sensor barriers. Regarding craving management barriers, clients reported that manually logging cravings in real time was difficult, repeat notifications about cravings reminded them of their craving, and difficulty in integrating it into their daily digital routines impaired utilization to some degree.

Clients and providers identified similar barriers while using the app and sensor. Specifically, they mentioned connectivity issues between the app and the sensor, which were more common with Android users. They found the impact on phone battery life to be more prominent with lower-cost smartphones (e.g. using the pared-down “Android Go” operating system). Additional issues included: forgetting to charge the sensor, forgetting to put the sensor back on after charging, and having to wear an additional device outside of their personal smartwatch.

“…maybe just to… have it not necessarily [have to] run in the background at all times but activate only during some sort of stress or craving event.”

– 34-year-old male in recovery from opioid use disorder

“It is important that it is user-friendly, especially for those who are not technologically alert.”

– 59-year-old male in recovery from alcohol use disorder

Both clients and providers cited notifications that were mistimed as substantial barriers: alerts that occurred while driving or sleeping were bothersome regardless of accuracy. They noted that there was a notification threshold frequency where meaningful reflection turned into excessive reminders: once this threshold had been crossed, the tool changed from helpful to detrimental. Along these lines, clients reported that expectations for 24/7 use of the device and a 100 % response rate were unrealistic and added too much pressure to their daily lives. They advocated for flexible “prescriptions” for use that encouraged any use/response as opposed to penalization for an imperfect track record of use.

“I think that, you know, addiction, especially if it’s early, you struggle with everything. Every little thing can turn you around, and, you know, you want the least amount of distractions in your life.”

– 52-year-old female in recovery from alcohol use disorder

Location tracking via Global Positioning System (GPS) was controversial. Clients demonstrated “othering” when discussing GPS – namely, that GPS tracking may be necessary (or even desirable) for some people but not for them personally. Providers noted that GPS can be helpful but may be resisted by clients. They suggested that acceptance would vary based on the client’s stage of recovery, with clients early in recovery being more likely to reject the idea. They also noted that client acceptability would be influenced by the way the technology was presented: specifically, whether the technology was presented as an opportunity to (re)build trust and recovery capital, versus a tracking system.

“I think it also has to do with their stage of change…if they’re still pre-contemplative, the thought of having GPS on their phone [may be a barrier]. …Like today I’m gonna be sober but I don’t know next week. It might just be too much to accept in that moment of where they’re gonna be down the road.”

– Focus group provider

“This is how we’re going to build back with your family, your wife…It is never that we want to punish and track. Never.”

– Focus group provider (referring to GPS tracking in app)

### 3.5 Theme 4: desired features

Clients mentioned desired features for future integration into the app and sensor. For the app, they suggested: vital signs that they can view (e.g. heart rate), sleep and movement tracking, prediction of future stress/craving, additional provider collaboration, scheduled check-ins, and the ability to mute notifications.

“If it could alert you when …. you were stressed to breathe. Just because sometimes, you know you’re stressed out or you’re having a craving but you’re like…is that really stress….is that craving? …. It would help the person understand that your body is reacting to stress.”

– 41-year-old male in recovery from AUD and OUD

Providers desired more app customization, including modifiable text, features, organization of buttons/tiles, and appearance based on their preferences. They also mentioned client collaboration by adding features to track sobriety, meeting/appointment attendance, or other individualized goals over time (both SUD treatment and overall health-related). Gamification and/or social networking were desirable add-ons that providers felt would promote goal setting and reaching. Lastly, they wanted the app to integrate into their organizational electronic health record to avoid additional log-in requirements and minimize potential loss/fragmentation of information.

“The app should be customizable for both the clinician and the client.”

– Focus group provider

Regarding the sensor, both providers and clients desired a longer battery life, a larger screen, the ability to confirm or deny an event on the sensor screen, the ability to confirm or deny an event retrospectively, a calendar, talk-to-text, and different wristband materials. Additionally, they wanted integration with other devices (e.g. Apple Watch), which would allow for the synchronization of schedules, fitness, and other health-related items.

“…if it [sensor] was more like… part of like my Apple Watch. I think that would be better. [So that I] just have to wear that one thing, “

– 32-year-old male in recovery from alcohol use disorder

Providers requested the ability to monitor their client load and risk-stratify clients. They suggested an at-a-glance view to “see who is potentially most in danger,” and “what we need to act on now.” Managers would like to have a “progress bar” for the clinicians as a way for managers to ensure that the clinicians are responding to clients and that clinicians are not overwhelmed. Managers felt this system would be a tool to offer support to their clinicians, many of whom may be in recovery themselves.

## 4 Discussion

Thirty-one clients in recovery from SUDs and 11 outpatient SUD providers provided their perception and feedback surrounding a digital detection system for stress and craving. Overall, their reactions to the application and the wearable sensor were positive. Through client qualitative interviews and provider focus groups, we identified four major themes: experiences with stress and cravings, perceptions of both clients and treatment providers of a digital detection system during SUD recovery, identification of barriers and facilitators to adopting this new technology for SUD, and future desired features. Both groups agreed that the system was useful: for clients, it increased awareness and accountability, while for providers, it offered an opportunity to personalize treatment for a given client. There were some barriers to using the app that made it difficult for some to integrate the app into daily life.

The lower frequency of craving compared to stress has several potential explanations. It may stem from an actual lower frequency of craving, from challenges in quantifying craving, or from the societal stigma that discourages its open acknowledgment. The relationship between stress and craving also complicates the interpretation of their prevalence disparities. Multiple studies indicate that stress is associated with increased drug craving and risk of return to drug use (Sinha, 2008) and that patients with SUD often experience craving in response to stress and depression (Sinha, 2024).

A subset of participants reported that notification frequency and/or timing could be a barrier to use. While notifications for stress and craving were generally reported as helpful, some participants noted that notifications were repetitive (e.g. reminding them of the same stress or craving event that had not resolved) or inconvenient (e.g. occurring while driving or working). Prior research shows that designing an intervention dose is imperative to minimize harm and burden while promoting usefulness and optimization (McVay et al., 2019). As digital health detection becomes more common in the SUD population, future studies should focus on the optimal dose for digital interventions to identify key variables contributing to harm reduction versus harm creation.

Participants expressed a desire for the capability of tracking more than just craving and stress, including sleep, movement, location, interactive queries (ex: “How are you doing today?”), and individualized suggestions to help de-escalate (ex: “Go to the gym”, “Call your mom”) along with positive affirmations. Additionally, they hope future versions of the app and sensor will have the option to add notes to qualify events, text to talk, and share data with their treatment providers. Generally, requests focused on increasing app functionality into an “all-in-one” wellness tool. However, some participants would rather not have to interact with an app on their phone at all and preferred to have a more seamless and preferably automated system with the sensor alone.

### 4.1 Limitations

The small sample size of both providers and clients and limited racial, socioeconomic, and gender diversity make it difficult to generalize feedback, as these can increase the risk of unintentionally broadening the existing digital divide in mHealth interventions for SUD (Hampton et al., 2024). Participants were recruited from an outpatient treatment setting and thus might have missed important considerations for the application of this type of technology in inpatient/residential settings. Due to the qualitative nature of the study, there is a risk of social desirability bias where participants’ perceptions of what interviewers want to hear influenced their responses. We attempted to mitigate this bias through the use of facilitators who had no ownership or financial interest in the app, implementing best practices for qualitative interview techniques, and explicit statements at the start of each interview that all honest feedback (positive and negative) was helpful. Finally, in this small sample, we were unable to explore the complexity and heterogeneity of SUD with respect to substance type and stage of recovery, which likely play an important role.

There is a possibility of selection bias in the sample as just over 50 % of the participants in the parent study completed the semi-structured interview. Interview completers were more likely to be male, iOS users, and very early (< 30 days) or very late (> 1 year) in treatment. This likely influenced the conclusions gathered from our data, but also potentially provides clues to the demographic that we were able to keep engaged with the app. iOS users, for example, may reflect a higher income and education population and clients at the more extreme ends of the recovery spectrum may have unique needs.

### 4.2 Implications and plan for the future

A 42-year-old female in recovery from alcohol use disorder in the study noted that “addicts [sic] don’t like to admit when they need help”. Considering the commonality and complexity of stress and cravings during SUD recovery, digital health tools designed to objectively identify these key states could identify those moments when intervention is needed and extend the treatment milieu into the real world. Future work should specifically investigate the use of RAE in different substance use disorders, and different stages of recovery. Understanding end-user perceptions of digital health systems is critical for improving adoption, integration, and efficacy. The optimal dose of the digital intervention is also a critical factor that requires elucidation to ensure that the intervention brings value and not burden. Current ongoing trials of the RAE Health system include the use of a peer-based companion app that can be used by peer recovery coaches to follow client recovery and the use of craving biomarkers to measure outcomes in medications for opioid use disorder. These studies are piloting key features identified from our qualitative data, including strategies for reduced notification frequency, customizable interfaces, and device-agnostic operations.

## 5 Conclusions

A digital detection system for stress and craving during SUD recovery was perceived favorably by both clients and providers, with clients citing heightened awareness and providers citing opportunities for personalized care as promising use cases. Future iterations of digital health systems for this population should consider the ideal “dose” of the intervention to maximize benefit.

## CRediT authorship contribution statement

**Kapadia Nirzari:** Writing – review & editing, Writing – original draft, Formal analysis. **Eugene Reynalde:** Writing – review & editing, Writing – original draft, Data curation, Conceptualization. **Geagea Amanda:** Writing – review & editing, Writing – original draft. **Caggiano Emily:** Writing – review & editing, Writing – original draft. **Watson C James:** Writing – review & editing, Writing – original draft, Methodology, Investigation. **Carreiro Stephanie:** Writing – review & editing, Writing – original draft, Visualization, Validation, Supervision, Resources, Project administration, Methodology, Investigation, Funding acquisition, Formal analysis, Data curation, Conceptualization. **Hampton Jazmin:** Writing – review & editing, Writing – original draft, Formal analysis, Conceptualization.

## Funding

This was generously supported by NIH/NIDA (R44DA056162, MPI: Carreiro and R25DA058490, MPI: Carreiro).

## Declaration of Competing Interest

The authors declare that they have no known competing financial interests or personal relationships that could have appeared to influence the work reported in this paper.

## Footnotes

Appendix A Supplementary data associated with this article can be found in the online version at doi:10.1016/j.dadr.2025.100336.
