# Supplementary material for: Digital detection of craving and stress for individuals in recovery from substance use disorder: A qualitative study
Source: Drug Alcohol Depend Rep. 2025 Apr 19;15:100336. doi: 10.1016/j.dadr.2025.100336 (PMC12098147; doi:10.1016/j.dadr.2025.100336)
Supplement: Supplementary file 1 — Supplementary material [file mmc1.docx]

**Section 1: Intro**

*Intent: The goal of Section 1 is to explain the purpose of this interview and initiate the recording.*

- Thank the subject for taking part in our study.
- Inform the subject that we would like to hear about their experiences with the study. Any and all feedback they have is greatly appreciated.
- Explain that we will be recording this interview so we can gather more information from their feedback later.
  - Inform the subject that recorded data will only be labeled with a randomly generated Study ID# and that recording transcriptions will be scrubbed of identifying information.
  - Ask the subject to refrain from using their own name and real names of others during the recording.
- Explain they do not have to answer any questions you do not want to.
- Explain that some of these questions might be repetitive from the check in calls, but this is so we have it on recording and can go further in depth.
- Ask if they have any questions before we begin?
- Initiate the recording.
  - Turn on the Digital Voice Recorder and place it in a central location between yourself and the subject.
  - Begin the recording by stating the following information: *“Today is [DATE] and this is the RAE Exit Interview for [STUDY ID#]”.*
  - Re-ask the subject for permission to record the interview.

**Section 2: Perceptions of Stress and Craving**

*Intent: The goal of Section 2 is to explore subjects’ perceptions of their own stress and craving during the study period.*

- How has your recovery been since starting the study the study?
- How was your experience with stress during the study period?
  - How often did you experience stress?
  - What triggered stress?
  - Did you interact with the app when you felt stress?
- How was your experience with craving during the study period?
  - How often did you experience craving?
  - What triggered craving?
  - Did you interact with the app when you felt craving?

**Section 3: Perceptions on the use of the mobile app**

*Intent: The goal of Section 3 is to explore subjects’ perceptions regarding the use of technology. Specifically, we will focus on use of the mobile app in terms of intrusiveness, usability, improvements, and future research.*

- How was your experience using the RAE app?
  - Any issues or difficulties?
  - How often did you use it?
  - Do you normally keep your phone nearby you?
  - Did you keep the RAE app open in the background?
- Did the app accurately identify when you were stressed or craving?
  - How often was it accurate?
  - How often did it miss episodes that you felt (either stress or craving)?
  - How many notifications did you receive per day on average?
    - Did you respond to the notifications?
    - What were the barriers to responding to the notifications?
- When you experienced stress or craving, how did you interact with the app?
  - Did you ignore the notifications?
  - Did you complete any of the exercises?
  - Which features were most helpful?
  - Which features are least helpful?
- Did you discuss the data in your app (or clinical portal) with your treatment provider?
  - Probe: What data?
  - Probe: Did it affect your treatment plan? How?
- Did the app interfere with your daily life?
  - Probe: Did you find it annoying?
  - Probe: Were there too many prompts? Too few?
- What features would have made the app more useful?

**Section 4: Perceptions on the use of the Sensor**

*Intent: The goal of Section 4 is to explore subjects’ perceptions regarding the use of technology. Specifically, we will focus on use of the sensor in terms of intrusiveness, usability, improvements, and future research.*

- Can you tell me about your experience with the sensor?
  - Probe: What did you like about it?  What didn't you like about it?
  - Probe: Did you find it annoying to wear?
  - Probe: Did you have any problems using the sensor? Charging?
- Is there anything you did differently because you were wearing the sensor?
  - Probe: Anything you thought about differently?
- Did anyone react to you wearing the sensor?
  - Probe: Program Staff? Family? Friends? Strangers?
- Would you be willing to wear the sensor for a longer period of time?
  - Probe: How long?
- What features would have made the sensor more useful?

**Section 5: Subject Initiated Comments & Reflections**

*Intent: The goal of Section 5 is to allow subjects to provide any additional feedback.*

- Is there anything else that we did not cover that you would like to add?
  - Probe: Anything about the sensor? The app? The study in general?

**Section 6: Closing Remarks**

- Thank the subject for participating in this interview and inform them that you will now stop the recording.
- Turn off the Digital Voice Recorder.
- Answer any lingering questions.

**Section 7: Reflection**

*Intent: The intent of Section 7 is to write a general reflection about the interview that includes thoughts about the subjects’ attitudes, reactions, and any other potentially pertinent information. This should be done immediately after the interview, once you have left the subjects’ area.*

Study Staff Reflection:
