# Supplementary material for: Digital detection of craving and stress for individuals in recovery from substance use disorder: A qualitative study
Source: Drug Alcohol Depend Rep. 2025 Apr 19;15:100336. doi: 10.1016/j.dadr.2025.100336 (PMC12098147; doi:10.1016/j.dadr.2025.100336)
Supplement: Supplementary file 2 — Supplementary material [file mmc2.docx]

**RAE Health: Phase I**

**Initial Provider Focus Group Interview Guide**

*The intent of this focus groups is to understand the clinicians’ current needs in regard to the treatment program. We will be asking about their current problems, needs, and barriers and the ways that they think these needs can be filled by a wearable sensos, mobile app, and/or an online platform. Furthermore, we will demonstrate the RAE app and clinical portal and will allow clinicians to interact with them. We will then probe about their feedback of these models. This guide will serve as an outline but will not be adhered to verbatim.*

- Thank the clinicians for taking part in the focus groups
- Inform the clinicians that we would like to hear about their experiences with the treatment program and any problems/needs they have. Any and all feedback they have is greatly appreciated.
- Explain that we will be recording this interview so we can gather more information from their feedback later.
  - Inform the clinicians that recorded data will be deidentified and that recording transcriptions will be scrubbed of identifying information.
  - Ask the clinicians to refrain from using their own name and names of clients or other clinicians during the recording.
- Initiate the recording.
  - Turn on the Digital Voice Recorders and place it in a central location.
  - Begin the recording by stating the following information: *“Today is [DATE] and this is the RAE Initial Provider Focus Group at [Treatment Facility]”.*

Treatment Program Issues/Needs Assessment

- Please tell us about the current issues do you commonly encounter in the treatment program that threaten clients’ success.
  - Could any of these problems be addressed with a wearable biosensor/clinical portal? If so, how would you envision that helping?
- Do your clients ever express certain needs that are not being met?

RAE App (Demonstrate the App features and functionality)

- What is you first impression of the App?
- What barriers might your clients have with this App?
- What do you think of the:
  - Format/appearance?
  - Journal?
    - Any other questions you think we should ask?
    - Slider bar?
  - Breathe?
  - Contacts?
  - Geolocation feature/Check in feature
- Which features would be most useful? Least useful?
  - Why?
- Any additional features you/your clients would find helpful?

Integration into Practice

- Do you see this as a beneficial addition to current treatment protocol?
  - What potential barriers to you anticipate?
  - How difficult will this be to integrate into the current treatment protocol?
  - Would this change your practice in any way?
    - If so, how?
    - If not, why?
  - Any additional features you would make it easier to integrate?
