# Supplementary material for: Digital detection of craving and stress for individuals in recovery from substance use disorder: A qualitative study
Source: Drug Alcohol Depend Rep. 2025 Apr 19;15:100336. doi: 10.1016/j.dadr.2025.100336 (PMC12098147; doi:10.1016/j.dadr.2025.100336)
Supplement: Supplementary file 3 — Supplementary material [file mmc3.docx]

**RAE (Realize, Analyze, Engage) – A Digital Biomarker Based Detection and Intervention System for Stress and Craving During Recovery from Substance Use Disorder: Phase I**

PI: Stephanie Carreiro, MD, PhD

| **Code Number** | **Code Name** | **Code Description** |
| --- | --- | --- |
| **1.0** | **Stress** | ***Includes*: Participant experiences, the null**  ***Excludes*: stress in the app** |
| 1.1 | Timing and context | *Includes*: Frequency, amount  *Includes*: Triggers, situational/location, and other context, time of day, length of sobriety (as it relates to stress) |
| 1.2 | Impact on recovery |  |
| **2.0** | **Craving** | ***Includes*: Participant experiences, the null**  ***Excludes*: craving in the app** |
| 2.1 | Timing and context | *Includes*: Frequency,  *Includes*: Triggers, situational/location, and other context, time of day, length of sobriety (as it relates to craving) |
| 2.2 | Impact on recovery |  |
| **3.0** | **RAE** | ***Includes*: Anything about RAE as a whole** |
| 3.1 | Mobile app | Participant engagement with the app, participant opinion of app/features, notifications (exclude mentions of responding/not responding to stress and cravings as those are part of compliance) |
| 3.2 | Sensor | *Information about the physical sensor. Also include battery life of Sensor*  *Includes:* both Garmin and embrace sensors |
| 3.3 | Perceived usefulness | Of the RAE system as a whole, what stage of recovery the participant thinks RAE would be most useful, other uses of the RAE system, the null  *Exclude*: anything outside the scope of RAE. For example, step tracking), |
| 3.4 | Perceived ease of use | *Excludes*: null (code difficulties under barrier) |
| 3.5 | Barriers | *Includes*: Features that are missing; features that made it difficult to use; including compliance (double code); notification fatigue (how often and how much is too much), ~~stigma: If anyone reacted to the participant wearing the sensor or using the app, includes the null.~~  ~~Include: external and internal judgement (i.e. “I was scared to use that app because it thought I would be judged”)~~  Excludes the null |
| 3.5.1 | Stigma | Includes: If anyone reacted to the participant wearing the sensor or using the app, includes the null.  Include: external and internal judgement (i.e. “I was scared to use that app because it thought I would be judged”) |
| 3.6 | Desired features |  |
| 3.7 | Perceived accuracy | If they felt the notifications were accurate; false positives/negatives, etc. |
| 3.8 | Integration into care | If they talked to providers about RAE system; includes the null (if they said they did not talk about it) |
| 3.9 | Behavior/mindset change | *Includes:* If they changed behaviors or did anything differently because they were using RAE, includes the null |
| 3.10 | Compliance | *Includes:* App, sensor, or both and includes the null. Misuse or use not as directed. Includes use of Garmin app instead of RAE app. If they wore the sensor and used the app.  Includes: “the baseline” wear sensor for 30 days and keep app running in the background. Also responding/denying stress and craving notifications  Excludes more specifics or if they answered every prompt (beyond confirming/denying stress and cravings). This is more engagement. |
| 3.11 | Willingness | Willingness to continue using RAE or in the study in general, includes the null |
| **4.0** | **Stress and Craving** | Do not double code with code 1 or 2, include the null |
| 4.1 | Timing and context |  |
| 4.2 | Impact on recovery |  |
| **5.0** | **Othering** | Attributing an opinion or quality to another group. For treatment providers, would be aside from suggestions for their own clients  Excludes the null |
| **6.0** | **SUD and recovery** |  |
| 6.1 | Specific to Tx providers | The aware treatment providers who were enrolled. Perspective from a treatment provider. |
| 6.2 | COVID | Impact of covid on recovery or on other facets of life, includes the null. |
| 6.3 | Recovery | Includes: Sobriety and lapses in recovery, and other treatment modalities.  Excluding comments that are exclusively related to stress and craving or mentions of sobriety only in context of stress and craving. |
| 6.4 | Other technologies | Includes: Soberlink, headspace, etc.  Includes the null |
| **7.0** | **Illustrative quotes** |  |
| **8.0** | **Novel findings** |  |
| 8.1 | Digital literacy | Self-described ability to use technology |
| 8.2 | Digital equity | An advantage/disadvantage and social determinants of health such as education barriers, language barriers, race/ethnicity |
| 8.3 | Study protocol | Any suggested changes or improvements. Excludes include null. |
| **9.0** | **Other (Not sure how to code)** |  |
